# Clinical Value of Poor-Law Infirmaries

**Published:** 1915-02-06

**Authors:** A. G. Stewart

**Affiliations:** Medical Superintendent. Paddington Infirmary, Harrow Road, W.


### Clinical Value of Poor-Law Infirmaries.

*To the Editor of* The Hospital.

Sir,—It is fashionable for everyone to hit at the Poor-Law infirmaries, but the writer of the article in your issue of January 23 makes such sweeping statements that I feel that it is only fair of you to allow me to reply to them, as they most certainly do reflect on the work of the medical staff of these institutions. If your writer really believes, as he states, that "the fault is not with the individual officers," when he brings, amongst many others, a charge that "a case may remain for months with only the original prescribed treatment recorded," then all I can say is that his standard of duty is lower than mine.

Firstly, as regards records your writer says: "There are no continued records, no deliberate system, no definite scheme even in the most up to date of Metropolitan infirmaries." Records are kept here of all cases admitted to our infirmary, giving family history, previous history, history of present illness, present condition, and subsequent notes. (I enclose copy of our record papers.) The temperature charts, urine charts, massage charts, etc., are attached, and records of all bacteriological examinations made, *x*-ray examinations, etc., are attached. Every article of diet is written down, and the sisters are not allowed to give any treatment which is not prescribed on the notes. The notes in some cases occupy as many as six foolscap pages. Every quarter the notes are bound (male and female volumes), and a double index of patients' names and diseases made. Every morning a list of admissions for the previous day is brought to my office, with the date of last discharge in case of readmission (this is derived from the gate porter's book), and as a double check the sister asks each patient if he has been here before, and sends such record papers to my office. Every readmission is looked up, and volume and page with diagnosis, and any important particulars, such as operations, weight on discharge, presence of t.b.s. in sputum, etc., are written in the previous history space of fresh record papers.

In the case of patients admitted more than once during the same quarter the old notes are attached to the fresh history sheet. Apart from the medical value of this system, I can see if conduct of patient was in any way unsatisfactory on his previous visits, if he went to a convalescent home on discharge, and other useful details. I do not claim any merit in the matter, as this system has been in vogue since the Paddington Infirmary was opened thirty years ago. I shall be pleased to show our notes to anyone, and maintain that they are kept as fully and methodically as in many non-teaching voluntary hospitals, and certainly emphatically deny that they "are too spasmodic to be of any permanent value."

I am obliged to the writer for the note on *post-mortem*

records. Previously I have attached notes on the *post-mortem* result to the clinical notes, but in future I shall keep a separate register in addition. I have already prepared a scheme for indexing my $x$-ray plates.

As regards the annual report, a report from myself showing number of cases, methods of entry, ages, periods of stay in infirmary, notes on infectious diseases, tuberculosis, cancer, results of operations, etc., is now incorporated in the Guardians' annual report. In this matter your writer states: "The Metropolitan Asylums Board's medical institutions are an exception," etc. I have taken by chance the nearest volumes, and find that in 1912 the medical superintendent's report occupied: Fever hospitals, one case, two pages; in other nine cases an average of just over one page. Asylums, one case, eight pages; in other six cases, 1 to 6 pages. Two children's hospitals, three and four pages. I do not include statistics worked up at the Central Offices of the Board. The report of my colleague, Dr. Bayly, at Lambeth Infirmary for 1914 occupied thirty-two pages.

As regards the prejudice of the poor against the infirmary, I can give your writer the names of several doctors in our neighbourhood who have told me their poor patients prefer the infirmary to hospitals. I can also refer him to the clerk to the Guardians for my statement that patients have applied for admission to the infirmary, and have offered to pay the full cost of their maintenance.

As regards "the contempt of hospitals for infirmary work," it is difficult to answer your writer, as he refrains from giving the facts on which he bases this statement. Considering the disadvantages of the infirmary staff, such a contempt would be most unjust, the more especially when one remembers the many obligations the hospitals owe the staffs of the adjacent infirmaries. I recently showed a series of my operative results to a leading London Medical Society, from whose members, many on the staffs of voluntary hospitals, I have pleasure in stating I received every courtesy, and not the contempt your writer so freely bestows.—Yours faithfully,

A. G. Stewart, M.A., M.D.,
Medical Superintendent.

Paddington Infirmary, Harrow Road, W.,
January 30, 1915.

[We hope to publish next week the reply of the writer of the article.—Ed. The Hospital.]

